# Nanocantilevers with Adjustable Static Deflection and Significantly Tunable Spectrum Resonant Frequencies for Applications in Nanomechanical Mass Sensors

**DOI:** 10.3390/nano8020116

**Published:** 2018-02-17

**Authors:** Ivo Stachiv, Petr Sittner

**Affiliations:** 1 School of Sciences, Harbin Institute of Technology—Shenzhen Graduate School, Shenzhen 551800, China; 2 Institute of Physics, Czech Academy of Sciences, 18221 Prague, Czech Republic; sittner@fzu.cz

**Keywords:** NiTi film, shape memory alloy, phase transformation, nanocantilever, resonant frequency, static deflection, mass sensors, nanoresonator

## Abstract

Nanocantilevers have become key components of nanomechanical sensors that exploit changes in their resonant frequencies or static deflection in response to the environment. It is necessary that they can operate at a given, but adjustable, resonant frequency and/or static deflection ranges. Here we propose a new class of nanocantilevers with a significantly tunable spectrum of the resonant frequencies and changeable static deflection utilizing the unique properties of a phase-transforming NiTi film sputtered on the usual nanotechnology cantilever materials. The reversible frequency tuning and the adjustable static deflection are obtained by intentionally changing the Young’s modulus and the interlayer stress of the NiTi film during its phase transformation, while the usual cantilever elastic materials guarantee a high frequency actuation (up to tens of MHz). By incorporating the NiTi phase transformation characteristic into the classical continuum mechanics theory we present theoretical models that account for the nanocantilever frequency shift and variation in static deflection caused by a phase transformation of NiTi film. Due to the practical importance in nanomechanical sensors, we carry out a complete theoretical analysis and evaluate the impact of NiTi film on the cantilever Young’s modulus, static deflection, and the resonant frequencies. Moreover, the importance of proposed NiTi nanocantilever is illustrated on the nanomechanical based mass sensors. Our findings will be of value in the development of advanced nanotechnology sensors with intentionally-changeable physical and mechanical properties.

## 1. Introduction

Nanocantilevers have emerged as the fundamental components of micro(nano)-electromechanical systems (MEMS/NEMS), such as nanomechanical-based mass sensors [1,2,3,4,5]. It is the unprecedented physical and mechanical properties of materials in micro-(nano) dimensions that enable for the extraordinary high sensitivity and frequency stability. There are two different sensing principles of nanocantilever-based sensors. First, one utilizes the measurement of changes in a cantilever static deflection, i.e., the so-called “static mode” [6], whereas the second one enables evaluating the measured quantities from changes in the sensor frequency response, i.e., “dynamic mode” [7]. To determine the measured quantities, it is required that cantilever operates at given ranges of static deflection and resonant frequencies. Furthermore, for the dynamic mode, the cantilever must also exhibit a linear response to external stimuli [8,9,10] and, consequently, its sensitivity is then determined by the resonant frequencies [11].

In order to design these mechanical devices so that they can reach the required sensitivity and/or to keep the desired operating conditions during its lifetime, it is often necessary to compensate for changes in either the cantilever static deflection or the resonant frequencies that are caused by the fabrication tolerances and the unwilling environmental effects such as humidity and pressure [12]. For dynamic mode several methods capable of correcting the cantilever resonant frequencies have been proposed [12,13,14]. However, if we exclude the nanocantilevers made of extraordinary materials, like graphene or carbon nanotubes, then the common active resonant frequency tuning methods, such as piezoelectric or electrostatic ones allow usually only for a weak correction of the fundamental (first) resonant frequency, i.e., hundreds of Hz [9]. This is due to the fact that both of these methods mainly generate a relatively small in-plane stress on the cantilever surface, which causes only weak changes in the cantilever effective stiffness near its clamped end [15]. The impact of the mechanical stress on the resonant frequency strongly decreases with an increase of the vibrational mode [16,17]. Therefore, within a linear dynamic range, these two methods do not enable tuning of the resonant frequencies of higher vibrational modes. We must emphasize here that, in general, created in-plane stresses on the cantilever surface can also be large. For instance, the power intensity changes of the laser light used for probing the mechanical state of the cantilever resonator generates the in-plane surface stresses due to heating, which can result in large non-linear shifts of the cantilever resonant frequencies [18].

A low tunability of the nanocantilever resonant frequencies within the linear dynamic range limits their widespread use in high frequency operating linear sensors. Moreover, many other nanotechnology applications such as RF filters, power generators or amplifiers would benefit from cantilevers with significantly tunable spectrum of the resonant frequencies [19].

Nitinol (NiTi) is a metallic alloy that exhibits the unique functional properties like the shape memory effect and the superelasticity [20,21]. These properties are the result of a reversible diffusionless martensitic transformation driven by temperature and/or stress. The variation of both temperature and stress is involved in the shape memory effect, while the superelasticity involves only a stress-driven transformation [22,23,24]. The superelasticity is by far the most useful property, mainly in medical applications like stents [25]. There exist one-/two-way memory effects. The one-way shape memory effect represents a heating-induced reversal of shape of a deformed NiTi component in the absence of stress. The two-way memory effect the NiTi involves the memory of both low- and high-temperature shapes. The two-way memory effect is not an intrinsic property, but requires so-called “training” and, as such, the low-temperature strain in NiTi can easily become unstable [26]. The superelastic property of NiTi consists of the reversibility of a large deformation upon loading-unloading at constant temperature, i.e., up to ~5% for micro-/nanopillars and thin films [27]. It shall be pointed out that this large reversible deformation is a result of the reversible motion of phase interfaces during the phase transformation allowing the NiTi to return to the original shape after removal of the applied strain. The superelasticity can, thus, be exploited to generate the large flexural deflection of the NiTi cantilever vibrating at a constant temperature [21].

NiTi thin films have been conventionally used for microactuation in MEMS due to the high energy density involved [28]. Since the current NiTi actuators are set into motion by the martensitic transformation activated periodically by heating and cooling, the achievable actuating speed is low, just a few Hz, since thermal actuation also requires slow, natural cooling, which limits the achievable actuation frequencies [29,30]. As such, the majority of studies carried out so far on NiTi thin films focused on the variation of the NiTi film’s mechanical and physical properties with the slowly varying temperature and stress [31,32,33]. When temperature and stress varies quickly (>10 Hz), additional problems with the periodically-generated/absorbed latent heat and dissipated heat accumulation appear [34]. The heat exchange between the nanocantilever and environment affects the potential use of NiTi thin films in tunable high-frequency nanoresonators, such as ultrasensitive physical and chemical sensors or RF filters. However, as the displacement amplitudes in vibrating nanoresonators tend to be small, and heat exchange is fast, there is a good chance that the impact of the heat effect is not that critical, as in the case of bulky NiTi elements. To date, no systematic study on NiTi thin films for nanomechanical-based actuators and sensors have yet been performed.

In response to this challenge, we carried out the systematic theoretical investigations of nanocantilevers that take advantage of both the commonly-used nanotechnology elastic substrate materials, such as Si, SiO_2_, or Si_3_N_4_ (silicon nitride), and the NiTi alloys in a thin film form. A dedicated theoretical model that accounts for the variable elasticity of sputtered NiTi film and the interlayer internal stress between the film and the elastic substrate has been put forward. Then, based on the results, the impact of a phase-transforming NiTi film on the achievable changes in the cantilever effective Young’s modulus, static deflection, and the resonant frequencies is determined. The elastic substrate guarantees not only the high-frequency actuation (up to tens of MHz) but, due to a variation in the interlayer stress during a phase transformation of NiTi, it can also withstand non-negligible changes in static deflection [35]. The variable Young’s modulus of the NiTi film results in changes in the cantilever effective modulus and, correspondingly, in contrast to piezoelectric or electrostatic methods, it provides the extraordinarily high tunability of the multiple consecutive resonant frequencies of the designed nanocantilever. In addition to the unprecedented high frequency tunability, the NiTi nanocantilevers can be potentially used as sensors for multiple mass determinations, and for adsorbate molecule mass and stiffness measurements, as will be shown below. These examples will be of practical value to users of real-time nanomechanical-based mass sensors. Present theoretical models and analysis can also serve as a simple guide for further design of the nanomechanical sensors and actuators with changeable physical properties and tunable spectra of the resonant frequencies utilizing shape memory alloy (SMA) elements and films.

For the reader’s convenience, this paper begins with a brief introduction to NiTi film properties and NiTi nanocantilever preparation. In the second part of the paper a dedicated theoretical model accounting for a phase-transforming NiTi film is presented. In the third part of the paper, the role of a phase-transforming NiTi film on the effective Young’s modulus, static deflection and resonant frequencies of the designed NiTi nanocantilever is evaluated and, in the last part of the paper, examples of NiTi nanocantilevers for use as real-time mass sensors are given.

## 2. NiTi Film Preparation Methods and Properties

The NiTi films are prepared either by the vacuum magnetron sputtering method [23] or the bias target beam deposition method [36]. A uniform surface oxide layer independent of film thickness is naturally created on the free surface of a sputtered NiTi film. With a decrease of the film thickness, microstructure of the film changes and the oxide layer starts to affect its mechanical properties. Consequently, at film thicknesses of about 100 nm, the transformation behavior of NiTi starts to deviate from that known for bulk NiTi [27,32]. To prevent interdiffusion between the substrate and NiTi film, a thin interlayer made of Si_3_N_4_ must be deposited on the substrate prior sputtering the NiTi film. Furthermore, NiTi films sputtered at relatively low temperatures are amorphous. In order to obtain phase-transforming crystalline films, they have to be crystallized by suitable heat treatment [23,27]. This is done by heating the cantilever with sputtered NiTi film to a temperature of about 500 °C for several minutes. Alternatively, the NiTi films can be also produced by sputtering at high temperatures [37]. The thermal coefficient expansion (CTE) of NiTi is essentially higher than that of the commonly-used nanocantilever substrate materials; therefore, once the cantilever is cooled to room temperature a tensile stress is typically generated in the NiTi film upon cooling. This stress can be partially relaxed either if the NiTi starts to transform martensitically or the designed NiTi cantilever starts to bend upon cooling. The tensile stress in the NiTi film is balanced by the compressive stress in the substrate. Importantly, this internal stress in the cantilever can lead to a sharp decrease of the linear dynamic regime or to the non-linear vibration [38,8]. The low temperature sputtering followed by a heat treatment would be, therefore, the preferable route for most nanotechnology applications.

The density of the NiTi film is approximately 6.45 g/cm^3^ and does not change with temperature. However, the Young’s modulus of NiTi depends strongly on temperature. At low temperatures, NiTi film is in the martensite phase and the modulus ranges from 25 GPa to 40 GPa. Then, upon heating, the martensite starts transforming to an austenite phase and, correspondingly, the Young’s modulus increases. When martensite is fully transformed to the austenite, the Young’s modulus of NiTi reaches 60 GPa to 83 GPa. The martensitic transformation in 200-nm thick NiTi film sputtered on 200 nm thick Si_3_N_4_ substrate has been also observed just above room temperature, i.e., from 25 °C to 35 °C [32].

## 3. Theory

**Figure 1:**
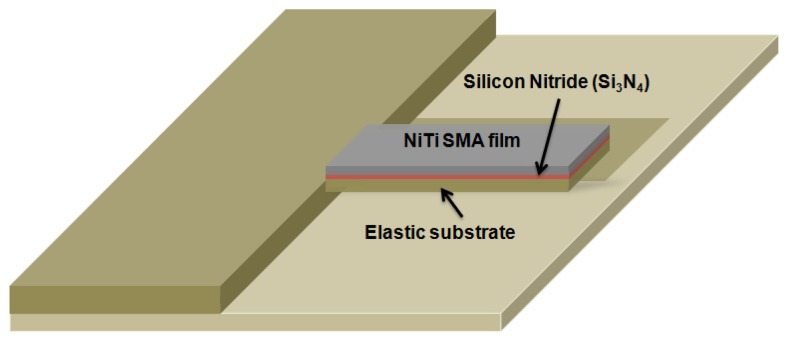
A schematic layout of NiTi/elastic substrate composite nanocantilever.

In this section, we present, based on the continuum mechanics theory and transformation characteristics of NiTi SMA, general theoretical models that enable accurate predictions of the static and dynamic deflections of a nanocantilever with a sputtered phase-transforming NiTi film. A schematic of the NiTi nanocantilever considered in this work is given in Figure 1. Despite the fact that the analysis is primarily carried out for NiTi films sputtered on Si, SiO_2_, or Si_3_N_4_ nanocantilevers, the obtained results are general and, hence, they are also valid for any other common nanotechnology used nanocantilever elastic materials. Densities and Young’s moduli of Si, SiO_2_, and Si_3_N_4_ considered in the present work are 2.33, 2.2, and 3.2 g/cm^3^, and 169, 69, and 310 GPa, respectively.

If temperature varies through the transformation range, the Young’s modulus of the NiTi film, as well as the interlayer stress between the film and substrate exhibits hysteresis, i.e., it is important to distinguish whether the current temperature was reached by cooling or heating [35]. This hysteresis, originating from the martensitic phase transformation, depends on the chemical composition, deposition, and annealing temperatures, etc. [20]. It shall be pointed out that the hysteresis width corresponding to cubic to monoclinic martensitic transformation is about 30–40 °C for common NiTi alloys. Fortunately, in the case of the NiTi thin film deposited on the elastic substrate, including the present case, the transformation range is extended and the physical properties vary with temperature with a small “effective” hysteresis of only a few degree Celsius [39]. This is the consequence of the constraint on film deformation/transformation exerted from the elastic constraint. In addition, the linear part of temperature dependency of the internal stress and Young’s modulus can differ for cooling and heating. In order to account for this in the present models, it is necessary to find dependencies of the Young’s modulus and interlayer stress on temperature for both parts of hysteresis experimentally. These measurements can be done, for instance, by the resonant methods [40] or nanoindentation [41]. In comparison to known piezoelectric or electrostatic tuning methods, where the in-plane stresses are generated mainly in a near vicinity of the cantilever clamped end [9], the interlayer internal stress originated from a phase transformation of NiTi film does create in-plane stresses over the entire cantilever length resulting in the static deflection and the axial loading *F_σ_* ≈ *σWT*_2_, where *W* is the cantilever width, *T*_2_ is the NiTi film thickness, and *σ* is the temperature-dependent average interlayer internal stress [42]. For ultrathin films deposited on a relatively thick elastic substrate *T*_2_ → 0 and *F_σ_* → 0, and, as expected, the surface stress dominates the cantilever response and the modified Stoney’s equation can be used to predict the cantilever deflection [43]. Nevertheless this equation does not account for the variable elasticity of the sputtered NiTi film; therefore, it cannot be, in principle, used to predict the static deflection of nanocantilever with the NiTi film of an arbitrary thickness. The required general dependency of cantilever deflection on the interlayer stress and the variable elasticity of NiTi film for various film thicknesses can be derived from a three-dimensional model by following the approach given in [44]. Shortly, at first a position of the neutral axis is determined. Then, a relationship between the normal stress and a curvature of considered two layered plate can be obtained, and after some simplifications and assumptions, e.g., stress in the film is isotropic and homogeneous, the desired expression-related bending of the cantilever with stress can be found. Now, omitting cumbersome calculations, the general relationship among stress, film elasticity, and the cantilever static deflection can be written as:

(1) $$z=\frac{3(1-\nu_{1})(1-\nu_{2})(1+\eta )\eta L_{}^{2}}{[(1-\nu_{2})+(1-\nu_{1})\xi \eta^{3}]E_{1}T_{1}}\sigma ,$$

where *z* is the temperature dependent “effective” cantilever deflection, *L* is the length of cantilever, *E* is the Young’s modulus, *T* is the thickness, *η* = *T*_2_/*T*_1_ is the thickness ratio, *ξ* = *E*_2_/*E*_1_ is the modulus ratio, *ν* is the Poisson’s ratio, and subscripts 1 and 2 stand for the elastic substrate and NiTi film, respectively.

For the vibrational amplitude that is much smaller than any of the cantilever length scale, the governing equation for the elastic deformation of the multilayered cantilever beam performing the flexural oscillations takes the following general form [45]:

(2) $$A_{1}\frac{\partial^{2}u(x,t)}{\partial t^{2}}+\left(A_{2}-\frac{A_{3}^{2}}{A_{4}}\right)\frac{\partial^{4}u(x,t)}{\partial x^{4}}\pm F_{T}\frac{\partial^{2}u(x,t)}{\partial x^{2}}=F_{\mathrm{ext}}(x,t).$$

Here $A_{1}=\sum_{i=1}^{N}\rho_{i}S_{i}$, $A_{2}=\sum_{i=1}^{N}E_{i}\int_{\Gamma_{i}}u^{*2}dS$, $A_{3}=\sum_{i=1}^{N}E_{i}\int_{\Gamma_{i}}u^{*}dS$, and $A_{4}=\sum_{i=1}^{N}E_{i}S_{i}$, *u*(*x*,*t*) are the dynamic deflection functions of the multilayered cantilever beam, *x* is the spatial coordinate along the length of the beam, *t* is the time, *N* is the number of the beam material layers, *S* is the cross-sectional area of the beam, *ρ_i_* and *E_i_* are the volumetric mass density and the Young’s modulus of the *i*-th material layer, u* is the local coordinate in the lateral direction, Γ*_i_* is the *i*-th region of the beam cross-section, ±*F_T_* stands for the resulting compressive/tensile axial load caused by the multiple physical and material effects, such as the residual stress, the CTE difference between each of the deposited material layer and, in our case, it also includes stresses originating from sputtering and a phase transformation of NiTi film, and *F*_ext_(*x*,*t*) is the external force per unit length, including driving and dissipative forces [46].

For nanocantilevers made of an elastic substrate and the sputtered phase-transforming NiTi film (see Figure 1) *N* = 2, *F_T_ ≈ F_σ_* is the tensile interlayer internal stress from NiTi [35,42], *A*_1_ = *W*[*ρ*_1_*T*_1_ + *ρ*_2_*T*_2_*H*], *A*_2_ = (*W*/12)[*E*_1_*T*_1_^3^ + (3*E*_1_*T*_1_*T*_2_^2^ + 3*E*_2_*T*_1_^2^*T*_2_ + *E*_2_*T*_2_^3^)], *A*_3_ = (*W*/2)*T*_1_*T*_2_(*E*_2_ − *E*_1_) and *A*_4_ = *W*[*E*_1_*T*_1_ + *E*_2_*T*_2_*H*]. It shall be pointed out that, for Si and SiO_2_ substrates, the interlayer film made of Si_3_N_4_ can be neglected since its thickness is essentially smaller than the thicknesses of the silicon and NiTi layers. The boundary conditions are the usual clamped-free end conditions with accounting for axial load:

(3) $$u(x,t)|{}_{x=0}=0,\frac{\partial u(x,t)}{\partial x}|{}_{x=0}=0,\frac{\partial^{2}u(x,t)}{\partial x^{2}}|{}_{x=L}=0,\left(A_{2}-\frac{A_{3}^{2}}{A_{4}}\right)\frac{\partial^{2}u(x,t)}{\partial x^{2}}|{}_{x=L}-F_{\sigma }\frac{\partial u(x,t)}{\partial x}|{}_{x=L}=0.$$

Solving Equation (2) with due account for the boundary conditions given by Equation (3) and *F*_ext_(*x*,*t*) = 0 yields the desired spectrum of the resonant frequencies:

(4) $$f=\frac{\gamma_{n}^{2}}{2\pi L^{2}}\sqrt{\frac{E_{1}T_{1}^{2}r(\xi ,\eta )}{12\rho_{1}(1+\mu \eta )}}.$$

Here, *r*(*ξ*,*η*) = [*ξ*^2^*η*^4^ + 4*ξη*(1+ 1.5*η* + *η*^2^) + 1]/(1 + *ξη*) *μ* = *ρ*_2_/*ρ*_1_ and *γ_n_*^2^ are the dimensionless resonant frequencies obtained as the positive roots of:

(5) $$\left(1+\frac{b^{4}}{2q_{1}^{2}q_{2}^{2}}\right)\cosh q_{1}\cos q_{2}+1+\frac{b^{2}}{2q_{1}^{}q_{2}^{}}\sinh q_{1}\sin q_{2}=0,$$

where $q_{1,2}=\sqrt{\pm \frac{b^{2}}{2}+\sqrt{\frac{b^{4}}{4}+\gamma^{4}}}$ and *b* ≈ $(L/T_{1})\sqrt{12\sigma \eta /(E_{1}r(\xi ,\eta ))}$ is the stress induced “tension parameter”, which accounts for the temperature-dependent average interlayer internal stress variation caused by the sputtered phase-transforming NiTi film.

## 4. Results and Discussion

### 4.1. Impact of Phase-Transforming NiTi Film on the Cantilever Effective Young’s Modulus and Static Deflection

In this section, we use the above theory to examine the impact of phase-transforming NiTi film on the effective Young’s modulus and static deflection of the NiTi nanocantilever. To begin, we note that the term (*A*_2_ − (*A*_3_^2^/*A*_4_)) in Equation (2) stands for the flexural rigidity of the multilayered cantilever that, for a two layered nanocantilever, i.e., NiTi film sputtered on elastic substrate, yields:

(6) $$D_{F}=(1/12)WT_{1}^{3}E_{1}r(\xi ,\eta ).$$

The flexural rigidity of any rectangular beam is given as (1/12) *WT*^3^*E* [47], thus, the effective Young’s modulus of the cantilever with accounting for variable elasticity of a phase-transforming NiTi film cannot be directly retrieved from Equation (6). However, by introducing the following dimensionless thickness parameters *h*_1_ = *T*_1_*/T*_T_, *h*_2_ = *T*_2_/*T*_T_ and *T*_T_ = *T*_1_ + *T*_2_ Equation (6) can be rewritten in the following way:

(7) $$D_{F}=(1/12)WT_{T}^{3}E_{1}r(\xi ,\eta )/T_{1}^{3}.$$

Now, as it is evident from structure of Equation (7), the term (1/12)*W*$T_{T}^{3}$ is nothing other than the moment of inertia of the nanocantilever and, consequently, the term *E*_1_*r*(*ξ*,*η*)/*T*_1_^3^ can be viewed as the effective Young’s modulus of the nanocantilever with sputtered phase-transforming NiTi film, which can be expressed as:

(8) $$E_{\mathrm{eff}}=\frac{E_{1}^{2}h_{1}^{4}+E_{2}^{2}h_{2}^{4}+4E_{1}^{}E_{2}^{}h_{1}^{3}h_{2}^{}+6E_{1}^{}E_{2}^{}h_{1}^{2}h_{2}^{2}+4E_{1}^{}E_{2}^{}h_{1}^{}h_{2}^{3}}{E_{1}^{}h_{1}^{}+E_{2}^{}h_{2}^{}}.$$

The variable elasticity of sputtered NiTi film related to its martensitic transformation yields changes in the cantilever effective Young’s modulus, which exact value depends on the substrate and film thicknesses and corresponding moduli. It shall be pointed out that the martensitic transformation of NiTi film does not change the Young’s modulus of the cantilever elastic substrate. Hence, it is useful for a further analysis of the one to consider variations in the cantilever effective Young’s modulus as its ratio to a known substrate Young’s modulus:

(9) $$E_{\mathrm{eff}}/E_{1}=\frac{1+\xi_{}^{2}\eta_{}^{4}+4\xi \eta (1+1.5\eta +\eta^{2})}{\left(1+\xi \eta )(1+\eta^{3}\right)}.$$

**Figure 2:**
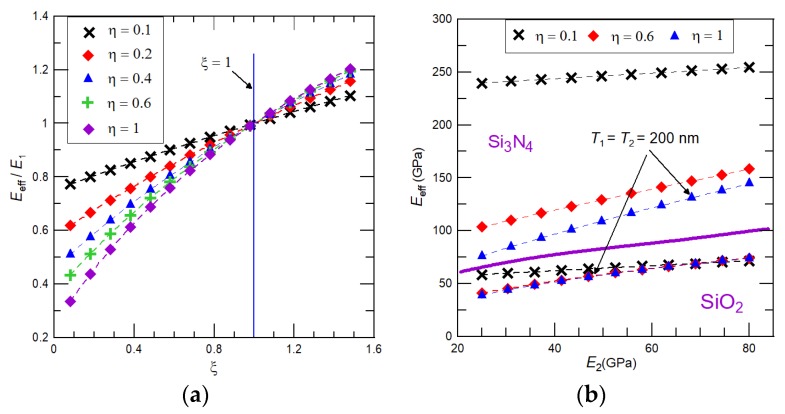
(**a**) Variation of the relative effective Young’s modulus of the cantilever, i.e., *E*_eff_/*E*_s_, as a function of the temperature-dependent NiTi film Young’s modulus represented through the dimensionless modulus ratio *ξ* for different thickness ratios *η*; and (**b**) the cantilever effective Young’s modulus as a function of the NiTi thin film modulus for two different substrate materials, SiO_2_ and Si_3_N_4_.

The relative changes in cantilever Young’s modulus depends on the combination of temperature-dependent dimensionless modulus parameter *ξ* and the film to substrate thickness ratio represented by *η*. Equation (9) discloses that the modulus variation depends strongly on film thickness (as *η*^4^) and less significantly on the modulus ratio (as *ξ*^2^). Hence, it can be expected that, for ultrathin films, the impact of the NiTi phase transformation on the cantilever effective Young’s modulus is negligibly small. Indeed, results presented in Figure 2a reveal that, for *η* ≤ 0.2, *E*_eff_ depends linearly on the dimensionless modulus ratio *ξ*. As such, the variation of cantilever effective modulus can be considered to be almost independent on the martensitic transformation in NiTi film. Nevertheless, with a further increase of film thickness, the impact of NiTi phase transformation starts to notably affect the effective Young’s modulus. For 0.2 < *η* < 0.5 the quadratic dependency and for 0.5 ≤ *η* the cubic dependency of the cantilever effective Young’s modulus are found. It is important to note that with an increase of substrate stiffness changes in the dimensionless modulus parameter *ξ* decreases and, consequently, for a given thickness ratio *η*, the dependency of *E*_eff_ on NiTi film during its phase transformation is almost linear, but cannot be neglected. For illustration, dependencies of the cantilever effective Young’s modulus as a function of the variable NiTi film modulus for two different substrate materials SiO_2_ and Si_3_N_4_, and three different film-to-substrate thickness ratios 0.1, 0.6, and 1 are shown in Figure 2b. As expected for Si_3_N_4_, almost linear dependency of *E*_eff_ on the NiTi film modulus is observed for all considered values of *η*, while, for SiO_2_, the linear dependency is realized only for *η* = 0.1; for the SiO_2_ substrate and *η* = 0.6 and 1, the cubic dependency is found. Figure 2b also reveals that, for a given film thickness, essentially larger variation of the cantilever effective Young’s modulus can be reached by sputtering NiTi film on stiff substrate materials, like Si_3_N_4_. For *η* = 1, e.g., a cantilever of *T*_1_ = *T*_2_ = 200 nm, the achievable changes in the cantilever effective Young’s modulus due to a phase transformation of the NiTi film are, for Si_3_N_4_ and SiO_2_ substrates, 70 GPa and 40 GPa.

Heating of the NiTi film is essentially faster than cooling [20,21], therefore, to set up the desired operating conditions by heating would be preferable for the majority of the nanotechnology applications. As such, we use the martensite phase in the NiTi film as the NiTi cantilever initial state. Then, the relative change in the cantilever deflection, accounting for variations in the interlayer stress and elasticity during a phase transformation of NiTi film from martensite to austenite, obtained from Equation (1) reads:

(10) $$z/z_{m}=\frac{(1-\nu_{2})+(1-\nu_{1})\xi_{m}\eta^{3}}{(1-\nu_{2})+(1-\nu_{1})\xi \eta^{3}{}_{1}}\frac{\sigma }{\sigma_{m}},$$

where subscript m stands for the NiTi film in the martensite phase.

**Figure 3:**
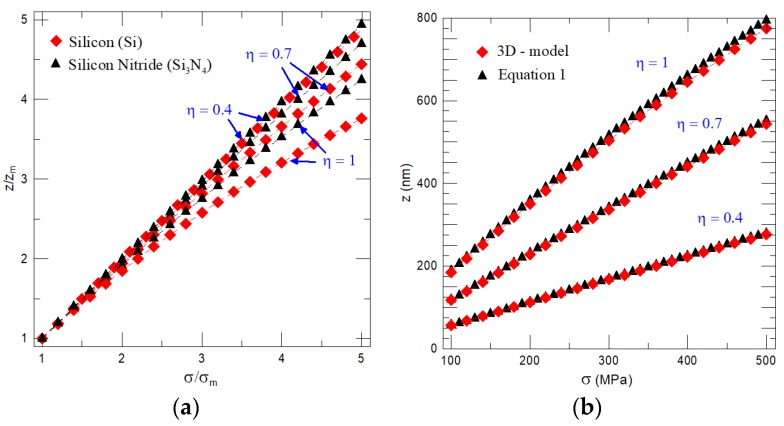
(**a**) Change in relative deflection *z*/*z*_m_ as a function of the temperature dependent tensile interlayer stress for different thickness ratios *η*; (**b**) estimated static deflections of the cantilever made of Si_3_N_4_ of *L* = 6 μm, *W* = 900 nm and *T*_1_ = 250 nm for three different NiTi film thicknesses as a function of the temperature dependent interlayer stress calculated by Equation (1) and the 3D model.

As can be seen from Equation (10), the cantilever deflection depends linearly on the interlayer stress and it is inversely proportional to the combination of NiTi film thickness and Young’s modulus as (*ξη*^3^)^−1^. Consequently, it can be easily concluded that for thin films, i.e., *η* << 1, changes in the static deflection of cantilever depend only on variation in the interlayer stress, i.e., *ξη*^3^ → 0. Dedicated calculations presented in Figure 3a demonstrate that the assumption of “thin NiTi film” holds for a cantilever with *η* ≤ 0.4, whereas, for a cantilever with a thickness ratio *η* > 0.4, the variation in the NiTi Young’s modulus comes into play and results in a weak, but not negligible, decrease of the cantilever’s deflection. The achievable static deflection calculated by Equation (1) and the three-dimensional model (3D model) for the cantilever made of silicon nitride substrate of length 6 μm, thickness 250 nm, and width of 900 nm with sputtered NiTi film of three different thicknesses is given in Figure 3b. As expected, for *η* = 0.4, cantilever deflection depends only on the variation in the interlayer stress and with a further increase of NiTi film thickness, *η* = 0.7 and 1, the weak non-linear decrease of the achievable static deflection is observed. For given cantilever properties and dimensions, Equation (1) yields the upper limit of static deflection, i.e., deflection predicted by theory (Equation (1)), is higher than the one obtained by a 3D model. The discrepancy between theory and the 3D model is due to a “plate approximation” used to derive Equation (1) [47] and, as it can be expected, it decreases with a decrease of film thickness represented by a thickness ratio *η* (see Figure 3b). Note that theoretical predictions given in Figure 3a,b also agree qualitatively with experiments obtained by a curvature measurement method for NiTi thin films [48] and, correspondingly, it allows us to confirm the validity of the derived expressions (Equations (1) and (10)) used for general prediction of the cantilever static deflection caused by a phase-transforming NiTi film.

### 4.2. Impact of Phase-Transforming NiTi Film on the Cantilever Resonant Frequencies

Here we use the theoretical model to evaluate the effect of a phase-transforming NiTi film on the cantilever resonant frequencies, i.e., “dynamic deflection”. Accounting for Equation (4), the relative frequency shift of cantilever with sputtered phase-transforming NiTi film can be expressed as:

(11) $$\Delta f/f_{m}={\left(\frac{\gamma }{\gamma_{m}}\right)}^{2}\sqrt{\frac{r(\xi ,\eta )}{r(\xi_{m},\eta )}}-\;1,$$

where *f*_m_ and *γ*_m_ are the resonant frequencies of the *n*-th mode of the cantilever with NiTi film in the martensite phase and Δ*f* = *f* − *f*_m_. Equation (11) clearly shows that, for the cantilever, the resonant frequency shift depends directly on the variable elasticity of NiTi represented by *r*(*ξ*,*η*) and indirectly on the interlayer internal stress *σ* represented by the stress-induced tension parameter *b* (see the structure of Equation (5)). We notice here that the impact of stress on the resonant frequency shift decreases with an increase of the vibrational mode, whereas the variable elasticity of the NiTi film is independent of the considered vibrational mode [16]. As a result, it can be easily concluded that by sputtering a phase-transforming NiTi film on the common nanocantilever materials it is possible to significantly tune multiple consecutive resonant frequencies of the designed NiTi nanocantilever.

We now define the dimensionless parameters that would help us to examine the frequency response of the nanocantilever of an arbitrary material properties and dimensions with due account for sputtered phase-transforming NiTi film of arbitrary values of the interlayer internal stress and the Young’s modulus. As is evident from Equations (4), (5), and (11), the natural dimensionless parameters can be easily defined as follows:

(12) $$b_{0}=\frac{b}{b_{m}}=\sqrt{\frac{\sigma }{\sigma_{m}}\frac{r(\xi_{m},\eta )}{r(\xi ,\eta )}},\;E_{\mathrm{eff}\text{\_}0}=\frac{E_{\mathrm{eff}}}{E_{\mathrm{eff}\text{\_}m}}=\frac{r(\xi ,\eta )}{r(\xi_{m},\eta )},\;\sigma_{0}=b_{0}^{2}E_{\mathrm{eff}\text{\_}0}=\frac{\sigma }{\sigma_{m}}$$

**Figure 4:**
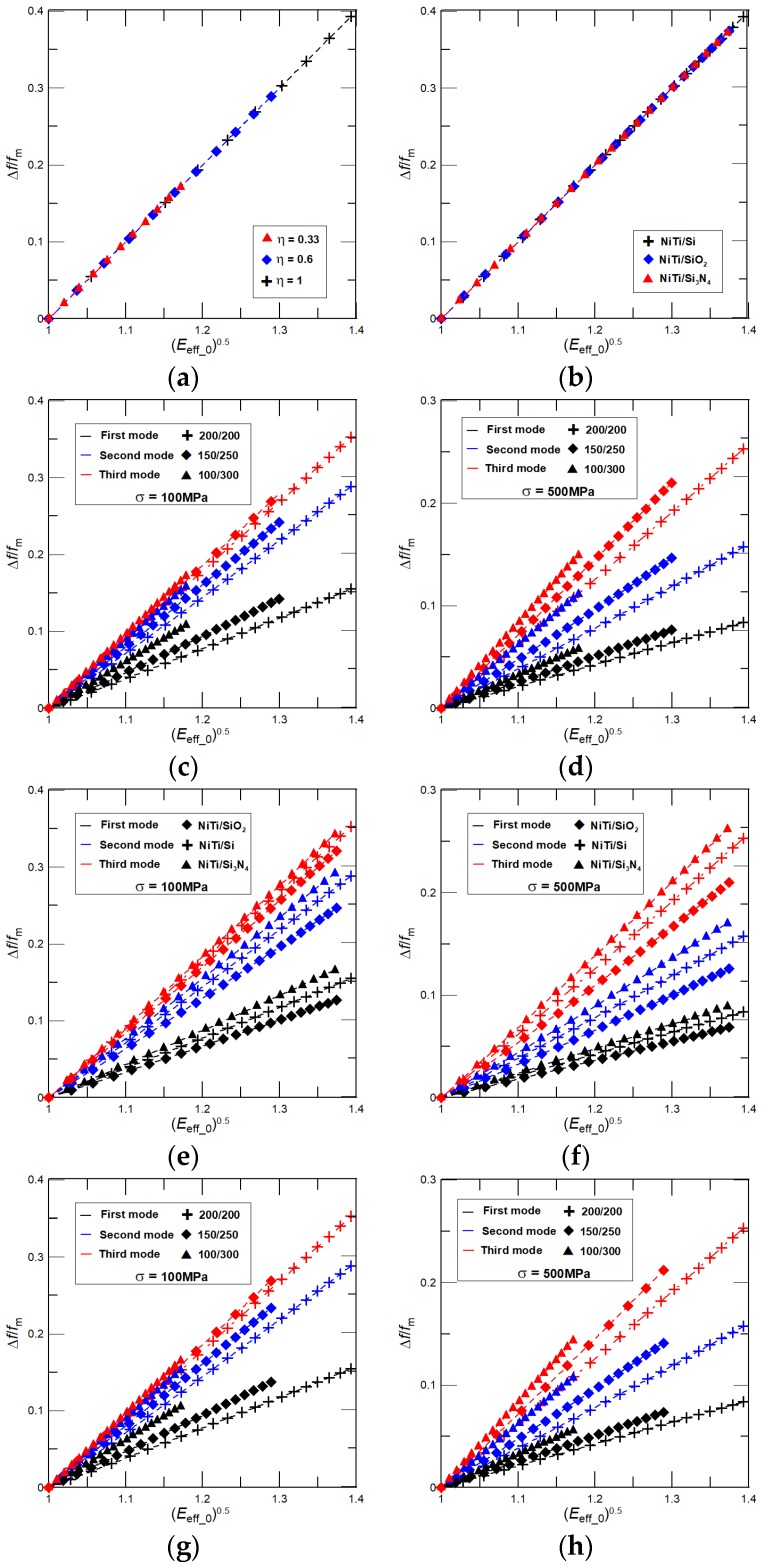
Relative frequency shift Δ*f*/*f*_m_ as function of (*E*_eff_0_)^0.5^ calculated for: (**a**) *σ* = 0 and a NiTi/Si nanocantilever of different thickness ratios *η* = 0.33, 0.6, and 1; (**b**) *σ* = 0 and NiTi/Si, NiTi/SiO_2_, and NiTi/Si_3_N_4_ nanocantilevers of *η* = 1. Both results show independency of Δ*f*/*f*_m_ on the cantilever geometry and properties (as predicted by theory). The dependence of Δ*f*/*f*_m_ on (*E*_eff_0_)^0.5^ by Equation (11) for the NiTi/Si nanocantilever of *η* = 0.33, 0.6, and 1, and (**c**) *σ* = 100 MPa, and (**d**) *σ* = 500 MPa. The dependence of Δ*f*/*f*_m_ on (*E*_eff_0_)^0.5^ was calculated by Equation (11) for NiTi/Si, NiTi/SiO_2_, and NiTi/Si_3_N_4_ nanocantilevers of *η* = 1 and (**e**) *σ* = 100 MPa, and (**f**) *σ* = 500 MPa. Δ*f*/*f*_m_ as a function of (*E*_eff_0_)^0.5^ for the NiTi/Si nanocantilever of *L* = 10 μm, *W* = 900 nm, and *η* = 0.33, 0.6, and 1 predicted by FEM and (**g**) *σ* = 100 MPa, and (**h**) *σ* = 500 MPa.

From Equations (11) and (12) it can be concluded that if a phase transformation of NiTi film is realized without any interlayer stress, i.e., *σ* = 0, then the relative frequency shift is independent of the considered vibrational mode, as well as the cantilever length, width, and density. The relative frequency shift would be just linearly proportional to changes in the cantilever effective Young’s modulus caused by a variable elasticity of NiTi film as *E*_eff_0_^1/2^. For a further analysis we consider only a linear part of the hysteresis with the NiTi film Young’s modulus ranges from 25 (martensite) to 80 (austenite) GPa. We note here that the identical linear increase of the NiTi Young’s modulus was used previously for the estimation of *E*_eff_ (see Figure 2b). Figure 4a,b present results for the relative frequency shift as a function of *E*_eff_0_^1/2^ calculated by Equation (11) for *σ* = 0 and: (i) a NiTi/Si nanocantilever of three different lengths *L* = 6, 8, and 10 μm and three thickness ratios, *η* = 0.33, 0.6, and 1, i.e., *T*_1_ = 300 (*T*_2_ = 100), 250 (150), and 200 (200) nm (see Figure 4a); and (ii) NiTi/Si, NiTi/SiO_2_, and NiTi/Si_3_N_4_ nanocantilevers of *L* = 6, 8, and 10 μm and *η* = 1, i.e., *T*_1_ = *T*_2_ = 200 nm. The width of all considered cantilevers is 900 nm. As it is predicted by the model, a general linear dependency of Δ*f*/*f*_m_ on *E*_eff_0_^1/2^ independent on the considered vibrational mode, cantilever size, and properties is obtained. Figure 4a also shows that, in accordance with theory, the achievable relative frequency shift increases with an increase of *η*.

If there is an interlayer internal stress in the prepared NiTi nanocantilever that, however, does not significantly vary during the phase transformation of the NiTi film, i.e., *σ* ≠ 0 and *σ* ~ constant, then, for the higher vibrational modes, a significant increase of the achievable frequency shift Δ*f*/*f*_m_ is found as shown for the NiTi/Si nanocantilever of *η* = 0.33, 0.6, and 1, and a low interlayer internal stress, i.e., *σ* = 100 MPa, in Figure 4c and for a large stress value, i.e., *σ* = 500 MPa, in Figure 4d, and also for NiTi/Si, NiTi/SiO_2_, and NiTi/Si_3_N_4_ nanocantilevers of *T*_1_ = *T*_2_ = 200 nm in Figure 4e (*σ* = 100 MPa) and Figure 4f (*σ* = 500 MPa). Moreover, the following two additional key features are also evident from Figure 4c–f: (i) a linear dependency of Δ*f*/*f*_m_ on *E*_eff_0_^1/2^, and (ii) the achievable tunability of Δ*f*/*f*_m_ decreases (increases) with an increase of the interlayer internal stress value (the NiTi film thickness represented by *η*). Both of these features, as well as the significant increase of the achievable frequency shift Δ*f*/*f*_m_ for the higher vibrational modes, are related to the variable elasticity of the NiTi film. The Young’s modulus of NiTi film during its phase transformation increases causing a linear increase of the relative frequency shift on *E*_eff_0_^1/2^ as shown in Figure 4a,b. At the same time, for given constant values of *σ*, the tension parameter *b* decreases with an increase of the NiTi film Young’s modulus, i.e., *b* ~ (*σ*/*E*_eff_)^0.5^, *E*_eff_ > *E*_eff_m_ and *b*_m_ > *b*, resulting in a decrease of the dimensionless resonant frequencies *γ*^2^. For simplicity, we suppose that *b* causes a small decrease in the dimensionless resonant frequency, i.e., *γ* ≈ *γ*_m_ − *a*_1_*b*^2^, *a*_1_*b*^2^ << 1, where *a*_1_ is some constant, *γ*^2^ ≈ *γ*_m_^2^(1 − 2 *a*_1_*b*^2^/*γ*_m_) and (*γ*/*γ*_m_)^2^ ≈ 1 − 2*a*_1_*b*^2^/*γ*_m_. Hence, for a constant *σ* with an increase of the considered vibrational mode the dimensionless resonant frequency of cantilever with NiTi in martensite state *γ*_m_ also increases shifting (*γ*/*γ*_m_)^2^ to the higher values and, in a limiting case, *γ*_m_ → ∞, (*γ*/*γ*_m_)^2^ ≈ 1 and Δ*f*/*f*_m_ ~ *E*_eff_0_^1/2^. Now, for the same vibrational mode, the higher values of the interlayer internal stress cause a decrease of the relative frequency shift and, in a limiting case, *σ* → ∞ and (*γ*/*γ*_m_)^2^ → 0, yielding a sharp decrease of the achievable frequency shift Δ*f*/*f*_m_, as evident from Figure 4d or Figure 4f. A good agreement between the present theoretical predictions, i.e., the analytical model, and the finite element simulations (FEM) presented in Figure 4g,h has been also found. For example, for the NiTi/Si nanocantilever discrepancy between the analytical model and FEM results is within a few percent.

We must emphasize here that when NiTi is sputtered at high temperature followed by cooling to room temperature, the CTE mismatch between the film and substrate results in the tensile interlayer internal stress that decreases with an increase of temperature. At the same time, the martensite phase of NiTi will be moved back to the austenite causing an increase of stress. The interplay among these two stresses, as well as other interface stresses can result, for some configurations and temperature ranges, in a constant value of the resulting average internal interlayer stress [35].

**Figure 5:**
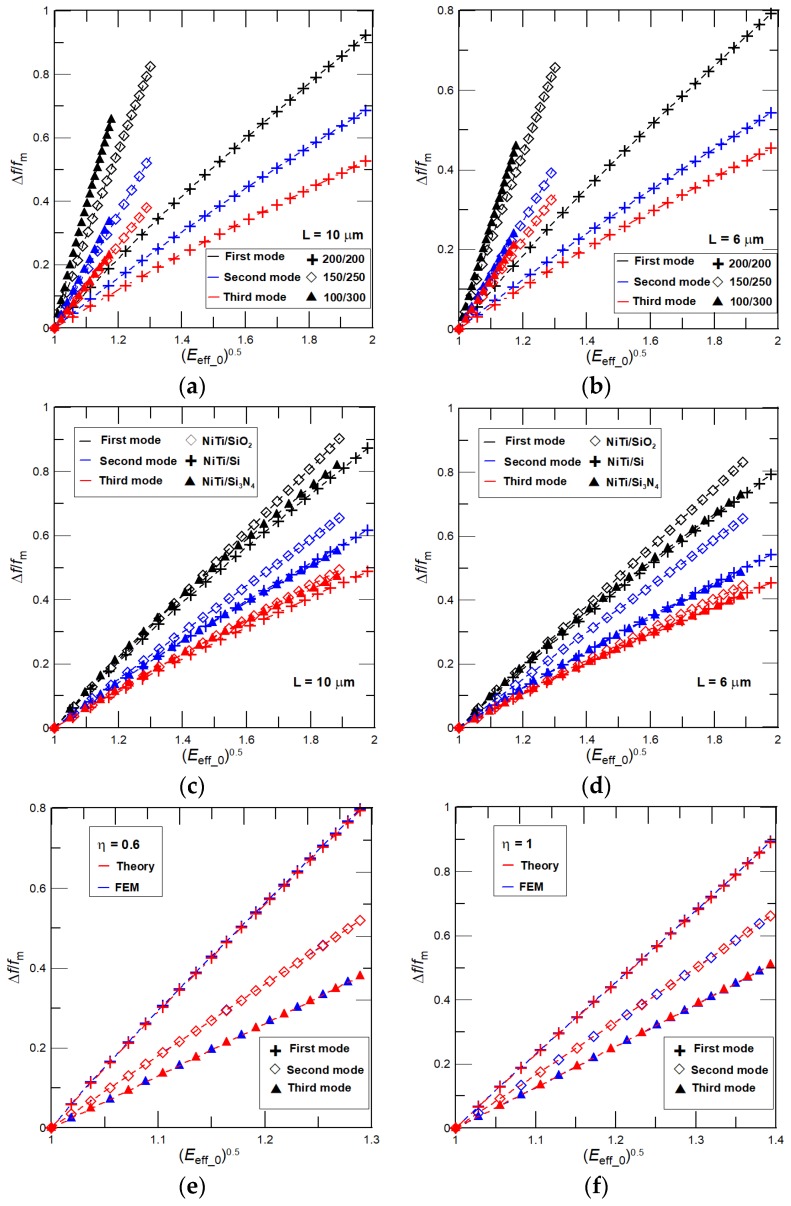
Relative frequency shift Δ*f*/*f*_m_ as function of (*E*_eff_0_)^0.5^ calculated by Equation (11) for NiTi/Si nanocantilevers of *η* = 0.33, 0.6, and 1, and length (**a**) *L* = 10 μm and (**b**) *L* = 6 μm. The dependency of Δ*f*/*f*_m_ on (*E*_eff_0_)^0.5^ for NiTi/Si, NiTi/SiO_2_, and NiTi/Si_3_N_4_ nanocantilevers of *η* = 1 and (**c**) *L* = 10 μm and (**d**) *L* = 6 μm. The dependency of Δ*f*/*f*_m_ on (*E*_eff_0_)^0.5^ obtained by the analytical model and FEM for NiTi/Si nanocantilevers of *W* = 900 nm, *L* = 10 μm and (**e**) *η* = 0.6 and (**f**) *η* = 1.

If NiTi is sputtered at a low temperature and followed by heat treatment, then the phase transformation of NiTi film causes an increase of both the cantilever effective Young’s modulus *E*_eff_ and the interlayer internal stress *σ*. As a result, the frequency shift Δ*f*/*f*_m_ depends on combination of a linear increase of *E*_eff_0_^0.5^ and a non-linear variation of (*γ*/*γ*_m_)^2^ that is proportional to the interplay between the stress induced parameter *b* and the effective elasticity *E*_eff_ (see Equations (11) and (12)). At first, let us consider that the phase transformation of the NiTi film is accompanied with a large linear variation of the interlayer internal stress ranging from 100 MPa (martensite) to 500 MPa (austenite) [32]. Then, for the considered nanocantilevers’ dimensions and properties, and ranges of the NiTi Young’s moduli and interlayer stresses, the dimensionless stress-induced tension parameter *b*_0_ = (*σ*_0_/*E*_eff_0_)^0.5^ increases with temperature from 1 (NiTi in martensite) to about 2.52 (NiTi in austenite), i.e., *σ*_0_, is from 1 to 5 and *E*_eff_0_ is from 1 to about 1.98 (for NiTi sputtered on Si substrate of *η* = 1). An increase of *b*_0_ shifts the dimensionless resonant frequencies *γ*^2^ to the higher values and, correspondingly, it enables the extraordinary high-frequency tunability of, particularly, the fundamental resonant frequencies, which is four times higher than the tunability obtained for a constant stress. Importantly, for higher vibrational modes, the impact of stress on the frequency response decreases resulting in the lowering of the achievable Δ*f*/*f*_m_ (see Figure 5). The higher frequency tunability can be also achieved by increasing the nanocantilever length. Additionally, an increase of *σ*_0_ is essentially faster than of *E*_eff_0_, thus, the weak non-linear dependency of Δ*f*/*f*_m_ on *E*_eff_0_^0.5^ can be observed as also shown in Figure 5.

**Figure 6:**
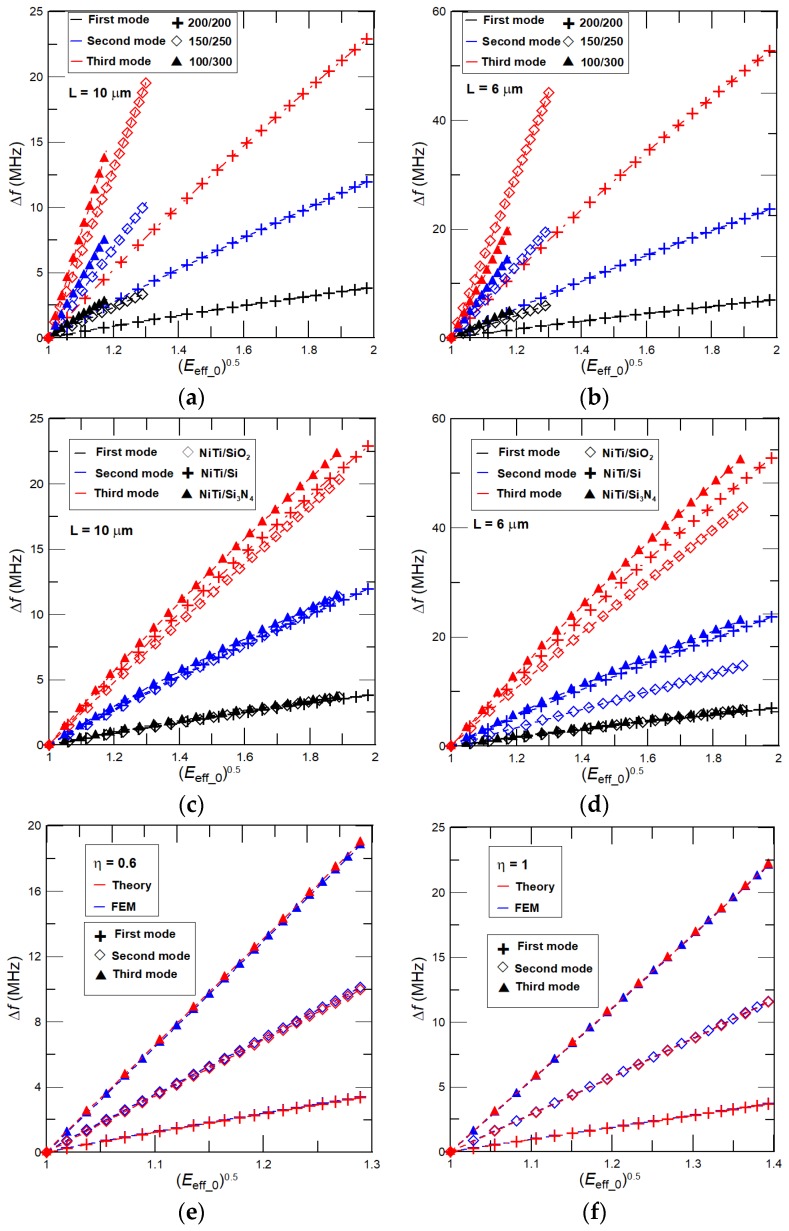
Absolute frequency shift Δ*f* as function of (*E*_eff_0_)^0.5^ calculated for NiTi/Si nanocantilever of *η* = 0.33, 0.6, and 1, and length (**a**) *L* = 10 μm and (**b**) *L* = 6 μm. Dependency of Δ*f* on (*E*_eff_0_)^0.5^ for NiTi/Si, NiTi/SiO_2_, and NiTi/Si_3_N_4_ nanocantilevers of *η* = 1 and (**c**) *L* = 10 μm and (**d**) *L* = 6 μm; *σ*_0_ = 1 to 5. Comparisons of Δ*f* on (*E*_eff_0_)^0.5^ predicted by present analytical model and FEM for NiTi/Si nanocantilevers of *W* = 900 nm, *L* = 10 μm, and (**e**) *η* = 0.6 and (**f**) *η* = 1.

In Figure 6 we present complementary results for the absolute frequency shift Δ*f* in nanocantilevers caused by the phase transformation of NiTi film. Here, we consider the identical nanocantilevers, stresses, and NiTi properties that have been used to analyze the effect of NiTi film on Δ*f*/*f*_m_. As expected, the absolute frequency shifts increase (decrease) with the considered vibrational mode (decrease with NiTi film thickness represented by *η*). For instance, for a nanoresonator of *L* = 6 μm and the NiTi film of phase transformation characteristics used in the present manuscript for analysis, the theoretically achievable extraordinary high-frequency tunability of the first resonant frequency of about 7 MHz is predicted by the present analytical model.

It is worth noting that for a nanomechanical resonator clamped at both ends, i.e., a suspended beam, the impacts of variable elasticity of NiTi and the interlayer stress on the resonant frequencies is essentially higher than for the cantilever beam case considered here. For example, for a nanoresonator of *L* = 6 μm, the tunability of 16 MHz (~30%) is calculated by an analytical model, i.e., for a doubly-clamped beam generated interlayer internal stress cannot be due to the clamped-clamped ends partially released and, as a result, its effect on the frequency shift is essentially stronger than the one for the nanocantilever. In addition, good agreements between resonant frequencies calculated by Equations (4) and (11), and predicted from the FEM simulations are found (see Figure 5e,f and Figure 6e,f). For NiTi/Si nanocantilevers of *η* = 0.6 and 1, the difference between Δ*f* obtained from FEM and the analytical model is within several percent. The resonant frequencies predicted by FEM are higher than those obtained by the present theory.

**Figure 7:**
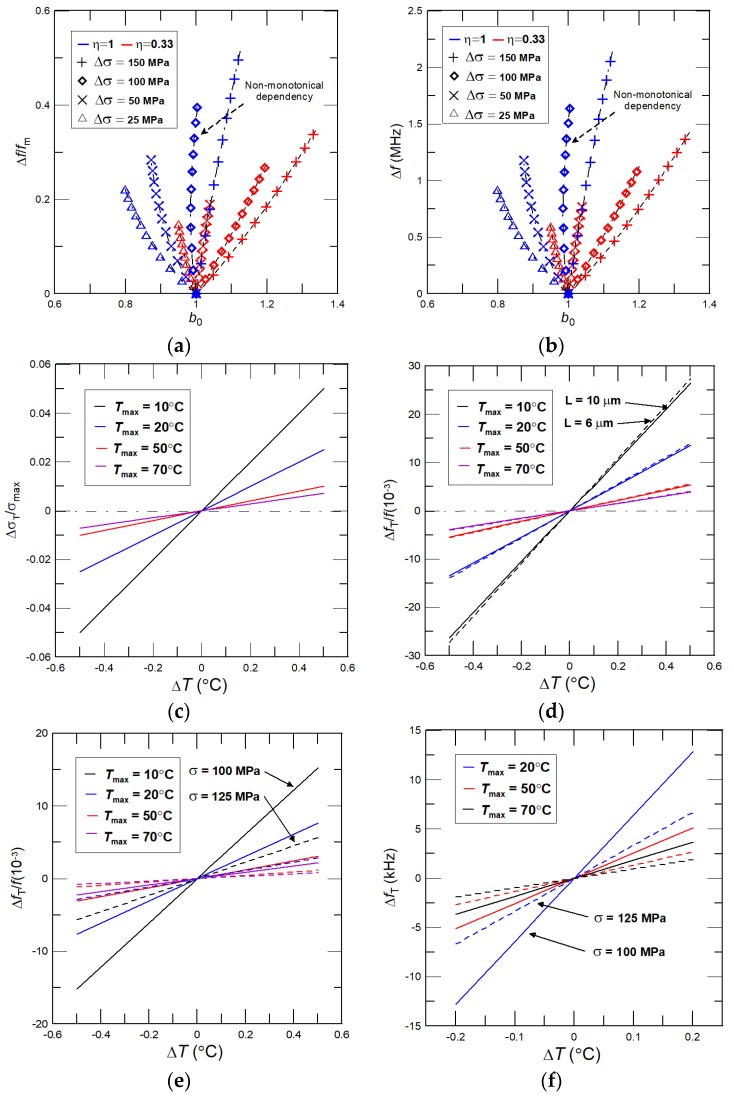
(**a**) Relative frequency shift Δ*f*/*f*_m_ and (**b**) absolute frequency shift Δ*f* as function of *b*_0_ for different moderate variations in the interlayer stress predicted for NiTi/Si nanocantilever of *L* = 10 μm, *η* = 0.33, and 1, and *σ*_m_ = 100 MPa; (**c**) dependency of the relative error in the interlayer internal stress Δ*σ*/*σ*_max_ on uncertainties in temperature Δ*T* for different NiTi transformation temperature ranges *T*_max_; dependency of error in the relative frequency shift Δ*f*_T_/*f* of the NiTi/Si nanocantilever on Δ*T* for different *T*_max_ for (**d**) *L* = 10 and 6 μm, *σ* = 100 MPa, and *σ*_max_ = 100 MPa, and (**e**) *L* = 10 μm, *σ* = 100, and 125 MPa, and *σ*_max_ = 25 MPa; (**f**) dependency of error in Δ*f*_T_ of the NiTi/Si nanocantilever of *L* = 10 μm, *σ* = 100, and 125 MPa, and *σ*_max_ = 25 MPa on Δ*T* for different *T*_max_; for all cases *σ*_m_ = 100 MPa.

Phase transformation of NiTi films that is accompanied with a large variation in the interlayer internal stress enabled us to evaluate the maximum theoretically-achievable resonant frequency tunability of the nanocantilever with due account for a phase-transforming NiTi film. Noticing here that during phase transformation of the NiTi film the moderate variation in the interlayer internal stress is often realized [28,35]. In general, the tension parameter *b*_0_ is proportional to the ratio of stress to the effective modulus variations as (*σ* + Δ*σ*)/(*E*_eff_0_ + Δ*E*_eff_0_), where Δ*σ* and Δ*E*_eff_0_ are the stress and modulus changes caused by a phase transformation of NiTi. For given changes in Δ*E*_eff_0_, the lower variation in Δ*σ* results in smaller values of *b*_0_ and, in a limiting case, Δ*σ* = 0 and *σ* = const., Δ*f*/*f*_m_ depends just on *E*_eff_0_^0.5^, and the considered vibrational modes are as shown previously in Figure 4c–f. Hence, for moderate variation of *σ*, the tension-induced parameter *b*_0_ can either weakly increase, decrease, or change non-monotonically. For the reader’s convenience, we present in Figure 7a,b the theoretically-achievable frequency shifts of Δ*f*/*f*_m_ and Δ*f* as functions of *b*_0_ for NiTi/Si nanocantilevers of *L* = 10 μm and thickness ratios of *η* = 0.33 and 1, *σ*_m_ = 100 MPa, and Δ*σ* = 150, 100, 50, and 25 MPa. The expected non-monotonic variation in *b*_0_ can be found for *η* = 1 and Δ*σ* = 100 MPa.

The NiTi films with high interlayer internal stress variation are of practical value for nanomechanical devices that are intended to operate at two significantly different resonant frequencies, i.e., “bistable” resonators. To ensure these resonators operate at desired values of the resonant frequencies, it is necessary that the NiTi film is either in the austenite or martensite phase. In this case, there would not be a shift in the cantilever resonant frequencies caused by a small variation of stress due to the unwilling temperature fluctuations. NiTi nanocantilevers with moderate interlayer stress can be used either to compensate for the frequency shift caused by drifting of the external temperature or in various actuators and sensors, where continuous tunability of the several consecutive resonant frequencies would be beneficial. In this case, the accuracy and application potential of these devices depends strongly on the NiTi phase transformation temperatures and uncertainties in the interlayer internal stress and NiTi elasticity that are caused by errors (fluctuation) in the setting of the temperature during the device operating conditions. To ensure that NiTi nanocantilevers with continuously-tunable resonant frequencies can be used in nanotechnology applications, we now examine the effect of uncertainties in temperature on the variation of the interlayer internal stress, NiTi elasticity and the nanocantilever fundamental resonant frequencies. For NiTi film sputtered at a low temperature, the interlayer stress and the NiTi Young’s modulus increase linearly with an increase of temperature during NiTi film phase transformation. Computations carried out over large ranges of the interlayer internal stresses and NiTi Young’s moduli, different transformation temperatures, and nanocantilever dimensions and properties reveal that there exists a linear dependency of a relative change in the interlayer internal stress and/or the NiTi Young’s modulus on the uncertainties in temperature setup as:

(13) $$\frac{\Delta \sigma_{T}}{\sigma_{\max}}=\frac{\Delta E_{2}}{E_{2\max}}=\frac{\Delta T}{T_{\max}}$$

where Δ*σ*_T_, Δ*E*_2_, and Δ*T* are the variations in NiTi stress, Young’s modulus and temperature, *σ*_max_ = *σ*_a_ − *σ*_m_, *E*_2max_ = *E*_a_ − *E*_m,_ *T*_max_ = *T*_a_ − *T*_m_, and subscript “a” stands for the NiTi film in the austenite phase. From Equation (13) we can conclude that, for a given inaccuracy in temperature due to thermal fluctuations, the lower error in *σ* can be achieved for NiTi films with a moderate and/or weak variation in the interlayer internal stress during its phase transformation or for NiTi film that the phase transformation is relatively slow, as given in Figure 7c. Noticing only that for common uncertainties in the temperature setting, the error in NiTi elasticity is negligibly small.

The interlayer internal stress does affect through the stress induced tension parameter *b* only the dimensionless resonant frequencies *γ*^2^ (see Equation (5)). As such, the inaccuracies in the temperature setup yield the errors in *σ* and, consequently, they cause small uncertainties in the cantilever resonant frequencies. If the error in stress Δ*σ* due to the temperature fluctuation Δ*T* is small, i.e., Δ*σ* << *σ* and Δ*E*_2_ ≈ 0, then Δ*b*/*b* ≈ 0.5 Δ*σ*/*σ*. For moderate interlayer stress, the dimensionless resonant frequencies calculated by Equation (5) can be accurately approximated as *γ*^2^ ≈ *γ*_B_^2^ + *a*_2_*b*^2^ + *a*_3_*b*, where *γ*_B_^2^ are the resonant frequencies without tension γ_B_^2^ = 3.516…, *a*_2_ = 0.2375, and *a*_3_ = 0.5547 [17]. Then, the error in the dimensionless resonant frequencies with due account for the error in Δ*σ* can be obtained as follows:

(14) $$\frac{\Delta \gamma }{\gamma }=\frac{1}{4}\left(\frac{2a_{2}b+a_{3}}{\gamma_{B}^{2}+a_{2}b^{2}+a_{3}b}\right)\frac{\Delta \sigma_{T}}{\sigma }$$

Equation (14) indicates that the error in desired frequency shift depends linearly on the uncertainties in temperature set up represented through the error in the interlayer internal stress Δ*σ*. Furthermore, the stress induced tension parameter is proportional to the interlayer internal stress and the cantilever length as *b* ~ *σ*^1/2^*L*, therefore, by increasing the cantilever length and/or stress, the parameter *b* also shifts to the higher values. At the same time, the denominator *γ*_B_^2^ + *a*_2_*b*^2^ + *a*_3_*b* in Equation (14) increases essentially faster than the numerator 2*a*_2_*b* + *a*_3_. As a result, for given uncertainties in the temperature setup, the lower error in the resonant frequencies can be easily achieved by increasing the cantilever length or utilizing the nanocantilevers with high initial values of interlayer internal stress, i.e., *σ*_m_ is high. To confirm these important results, we also perform computations for large ranges of the nanocantilevers’ dimensions, properties, interlayer internal stresses, different transformation temperature ranges, and uncertainties in Δ*σ*_T_ and Δ*E*_2_, and present the relative Δ*f*_T_/*f* and absolute Δ*f*_T_ frequency shifts for different inaccuracies in the temperature setup in Figure 4d–f. Importantly, these numerical computations are in good agreement with theoretical predictions obtained from Equations (13) and (14). For example, for a NiTi/Si nanocantilever of *η* = 1, i.e., *T*_1_ = *T*_2_ = 200 nm, of length *L* = 10 μm, with moderate variation in the interlayer internal stress ranging from *σ* = 100 MPa to *σ* = 200 MPa, the absolute frequency shifts Δ*f*_T_ (uncertainties in resonant frequency measurements) for *σ* = 100 MPa, Δ*T* = 0.01 and transformation temperature *T*_max_ = 20 °C (50 °C) are ~1.123 (0.45) kHz. Once stress reached the value of 200 MPa, then the error in frequency shift sharply decreases (almost two times) and for *T*_max_ = 20 °C (50 °C) Δ*f*_T_ ≈ 0.67 (0.27) kHz. For identical nanocantilever with stress ranging from 100 MPa to 125 MPa, Δ*T* = 0.01 and *T*_max_ = 50 °C the error in frequency determination of ~100 Hz can be easily achieved. Our results reveal that by using: (i) the higher initial stress values *σ*_m_; (ii) longer nanocantilevers; and/or (iii) increasing the transformation temperature ranges, the impact of thermal fluctuations on the nanocantilever resonant frequencies can be efficiently suppressed to values that are comparable with the usual errors obtained for piezoelectric and/or electrostatic frequency tuning methods [9]. These findings are of practical importance in the design of the nanocantilever-based sensors and actuators with significantly changeable spectra of the resonant frequencies utilizing the phase transformation of sputtered NiTi films.

For NiTi cantilevers designed at high temperature, the interplay between stress from CTE and stress originated from a phase transformation of NiTi operating around room temperature can also generate a non-monotonic variation in the interlayer internal stress resulting in the non-monotonic dependency of Δ*f*/*f*_m_ on temperature. In addition, despite the fact that the nanocantilever-based sensors and actuators utilizing the unique properties of phase-transforming NiTi film have not yet been experimentally studied, the validity of the general theoretical model represented by Equations (4), (5), and (11) were recently reinforced by experiments carried out on the microcantilevers with NiTi films sputtered at a high temperature [37]. In accordance with model, the expected non-monotonic dependency of Δ*f*/*f*_m_ on temperature were observed and Δ*f* increases with an increase of the considered vibrational mode. These experiments can, therefore, be used to verify the validity and robustness of the theoretical analysis and predictions carried out in the present work.

### 4.3. Nanocantilever Based Mass Spectrometry Utilizing the Phase Changeable NiTi Films

Using the above theoretical results, we now demonstrate the potential behind the NiTi nanocantilevers based devices. Particularly, we focus on the nanomechanical resonant based sensors, where the adsorbate mass(es) is (are) determined from changes in the cantilever frequency spectrum (see for example reviews [6,7]). Let us consider two commonly realized scenarios of chemical and biological adsorbate binding: (i) one or multiple adsorbate masses are attached to the cantilever resonator; i.e., for this scenario the total mass (sizes) of adsorbate molecules *m*_Σ_ (*d*_Σ_) is essentially smaller than the mass (dimensions) of the cantilever resonator *M* (*L*) [49]; and (ii) the adsorbate forms the uniform material layer on top of the nanocantilever [50].

For the first scenario the general method of multiple mass determinations by means of the axially-loaded cantilever resonators has been already proposed [11]. Briefly, each of the adsorbate masses do not change the cantilever stiffness, but do create an additional mass at a given location *h_j_*. To account for the frequency shift due to the adsorbates, the following matching conditions are imposed:

(15) $$\begin{array}{l} u(h_{i-},t)=u(h_{i+},t),\frac{\partial u(h_{i-},t)}{\partial x}=\frac{\partial u(h_{i+},t)}{\partial x},\frac{\partial^{2}u(h_{i-},t)}{\partial x^{2}}=\frac{\partial^{2}u(h_{i+},t)}{\partial x^{2}},\\ D_{F}\left(\frac{\partial^{3}u(h_{i-},t)}{\partial x^{3}}-\frac{\partial^{3}u(h_{i+},t)}{\partial x^{3}}\right)-F_{\sigma }\left(\frac{\partial u(h_{i-},t)}{\partial x}-\frac{\partial u(h_{i+},t)}{\partial x}\right)=m\frac{\partial^{2}u(h_{i},t)}{\partial t^{2}}. \end{array}$$

Here, subscript *i* stands for *i*th—attached mass and the subscripts + and − stand for the mass location to the direction of the free and clamped ends, respectively. Then, solving Equation (2) for boundary and matching conditions given by Equations (3) and (15) yields the following transcendental equation:

(16) $$\left(1+\frac{b^{4}}{2q_{1}^{2}q_{2}^{2}}\right)\cosh q_{1}\cos q_{2}+1+\frac{b^{2}}{2q_{1}^{}q_{2}^{}}\sinh q_{1}\sin q_{2}-\frac{1}{2}\frac{q_{1}^{5}B_{0}}{B_{1}q_{2}^{2}}\sum_{i=1}^{N}\varepsilon_{i}\sum_{j=1}^{m_{\Sigma }}H(q,h_{j})=0,$$

where *ε* = *m*/*M* and *H*(*q*,*h*) = *B*_2_^4^*G*_0_(*qh*) + *B*_2_^3^{cosh *q*_1_*h* sin [*q*_2_(*L* − *h*)] cos *q*_2_ + sinh *q*_1_*h* sinh [*q*_1_(*L* − *h*)] sin *q*_2_} + *B*_2_^2^{*G*_0_(*q*) − *G*_0_[*q*(*L* − *h*)] − cosh *q*_1_*h* cos [*q*_2_(*L* − *h*)] sin *q*_2_} + *B*_2_*G*_1_[*q*(*L* − *h*)] + *B*_2_^5^*B*_3_ sin *q*_2_{cos *q*_2_ + sin *q*_2_*h* sin [*q*_2_(*L* − *h*)]} + *B*_2_^4^*B*_3_{*G*_0_[*q*(*L* − *h*)] − (sinh *q*_1_ + sin *q*_2_) cos [*q*_2_(*L* − *h*)] cos *q*_2_*h*} + *B*_2_^3^*B*_3_{*G*_1_(*q*) + cosh [*q*_1_(*L* − *h*)] sin *q*_2_*h* cos *q*_2_} − *B*_2_^2^*B*_3_ {*G*_0_(*qh*) + sinh *q*_1_ sin *q*_2_*h* sin [*q*_2_(*L* − *h*)] + cosh [*q*_1_(*L* − *h*)] cos *q*_2_*h* sin *q*_2_} + *B*_2_*B*_3_ *G*_1_(*qh*) − cosh *q*_1_*h* cosh [*q*_1_(*L* − *h*)] sin *q*_2_ + (*b*/*q*_2_)^2^ {*B*_1_^2^[*G*_0_(*qh*) − sinh (*q*_1_*h*) cosh (*q*_1_(*L* − *h*)) cos *q*_2_] + *B*_2_[*G*_1_(*q*) + *G*_1_(*qh*) − *G*_1_(*q*(*L* − *h*))] + *B*_2_^3^*B*_3_[*G*_1_(*q*(*L* − *h*)) − cosh *q*_1_ cos (*q*_2_*h*) sin (*q*_2_(*L* − *h*))] + *B*_2_^2^*B*_3_[*G*_1_(*q*(*L* − *h*)) + *G*_1_(*qh*) − *G*_1_(*q*)] + *B*_2_*B*_3_[cosh *q*_1_ sin (*q*_2_*h*) cos (*q*_2_(*L* − *h*)) − *G*_1_(*qh*)] + cosh (*q*_1_*h*) sinh (*q*_1_(*L* − *h*)) cos *q*_2_ − *G*_0_(*q*(*L* − *h*))}, *B*_0_ = (*b*/*q*_1_)^2^ − 1, *B*_1_ = *q*_1_^2^ + *q*_2_^2^, *B*_2_ = *q*_2_/*q*_1_, *B*_3_ = [(*b*/*q*_1_)^2^ + *B*_2_^2^]/*B*_0_, *G*_0_(*z*) = sinh *z_1_* cos *z*_2,_ and *G*_1_(*z*) = cosh *z_1_* sin *z_2_*. Then, accounting for small mass ratio(s), i.e., *ε*_Σ_ << 1, the desired cantilever frequency shift due to *N* attached masses can be written in the following way [11]:

(17) $$\frac{\Delta f_{\mathrm{add}}}{f}=\frac{\gamma^{2}-\gamma_{\mathrm{add}}^{2}}{\gamma^{2}}=2\varepsilon_{\Sigma }\alpha_{\Sigma }(h_{j},\gamma ),$$

where Δ*f*_add_ = *f* − *f*_add_, *f*_add_ are the cantilever resonant frequencies with accounting for the adsorbate masses, *ε*_Σ_ = *m*_Σ_/*M* is a mass ratio accounting for attached masses, $\alpha_{\Sigma }(h_{j},\gamma )=\frac{1}{4}\frac{H(q,h_{j})}{B_{2}^{4}H_{D}(q,h_{j})}$ is the “mode shape function” and *H*_D_(*q*,*h*) = −2*b*^4^/(*B*_1_*B*_4_)·(1 − cosh *q*_1_ cos *q*_2_) + 2(2*q*_1_/*B*_1_ + *B*_1_/*B*_4_)·(cosh *q*_1_ cos *q*_2_ + 1) + [1 + b2/(2q22) + b4/(2q1B4)] sinh *q*_1_ cos *q*_2_ − [1/B2 − b2/(2q1q2) + b4/(2q2B4)] cosh *q*_1_ sin *q*_2_ + [*B*_1_/(*q*_1_*q*_2_*B*_4_) + 4/(*q*_2_*B*_3_)] sinh *q*_1_ sin *q*_2_ and *B*_4_ = *q*_1_*q*_2_^2^. Equation (17) enables one to decouple the effect of masses represented by *ε*_Σ_ and their positions of attachment given by *α*_Σ_(*h_j_*,*γ*). Once the adsorbate masses are attached to the nanocantilever surface their positions are given and do not change during measurements, and *α*_Σ_(*h_j_*,*γ*) depends only on the known dimensionless resonant frequency of the unloaded resonator as *γ*. Let us suppose only one attached mass on the nanocantilever. Then, two measured frequency shifts yield the following inequalities Δ*f*_add_F1_/*f*_F1_ = 2*εα*(*h*,*γ*_F1_) and Δ*f*_add_F2_/*f*_F2_ = 2*εα*(*h*,*γ*_F2_), subscripts F1 and F2 stand for two different allied axial force values. They results in [(Δ*f*_add_F2_/*f*_F2_)/(Δ*f*_add_F1_/*f*_F1_)] = *α*(*h*,*γ*_F2_)/*α*(*h*,*γ*_F1_) and for known *γ*_1_ and *γ*_2_ the position of mass attachment *h* is found. Plugging known values of *h* in Equation (17) and accounting for the one of the measured shifts, i.e., Δ*f*_add_1_/*f*_1_ or Δ*f*_add_2_/*f*_2_, the mass ratio *ε* and, consequently, the attached mass *m* can be determined. It is evident that to determine each adsorbate position of attachment and, afterward, the desired mass requires measurement of at least two resonant frequencies under different axial forces. In addition, it is a necessary condition that *α*_F1_(*h*,*γ*_F1_) varies significantly from *α*_F2_(*h*,*γ*_F2_). It has been shown that, to fulfill this important condition, requires *b* > 3, i.e., for *b* ≤ 3 the *α*(*h*,*γ*) does not notably change from the mode shape known for cantilever without axial load [11].

The usual electrostatic and piezoelectric tuning methods enable only a small correction of the cantilever fundamental resonant frequencies, therefore, for the same vibrational mode the mode shape function *α*(*h*,*γ*) can be considered constant and, as such, the method of multiple mass determinations by axially-loaded nanocantilevers does not work. Noticing only that for current nanotechnology cantilevers at least three consecutive resonant frequencies are needed to determine each of the adsorbate masses, limiting their usage to only either a single mass [49], or a couple of mass determinations in a real-time [51]. However, as one can expected, this serious drawback can be easily overcome by means of phase-changeable NiTi nanocantilevers. For example, for NiTi/Si nanocantilevers of *L* = 10 μm, *T*_1_ = *T*_2_ = 200 nm and W = 900 nm in the martensite phase, where *σ*_m_ = 100, 300, 500 MPa, the tension parameters *b*_m_ = 2.57, 4.45, and 5.74, while, for the austenite phase, where *σ*_a_ = 125, 325, and 525 MPa, i.e., a low interlayer internal stress variation of Δ*σ =* 25 MPa, *b*_m_ = 2.04, 3.29, and 4.18. As such, for *σ*_m_ (*σ*_a_) = 140 (270) MPa, *b* > 3 and the nanocantilever with sputtered phase-transforming NiTi film indeed enable multiple mass spectrometry in real-time. As one can expect, to determine *N* attached masses requires the measurement of *P* shifts of the fundamental resonant frequency of the NiTi film-based nanomechanical resonator, i.e., *P* ≥ 2*N*. The different shifts of the fundamental resonant frequencies are obtained by intentionally changing the effective Young’s modulus of the NiTi film and the corresponding interlayer stress between the NiTi film and the elastic substrate. It means that each frequency shift is measured for a given predetermined temperature that can be controlled by for instance thermostat. Interestingly, the weak hysteresis of NiTi film enables one to obtain two different frequency shifts for the same temperatures, i.e., the experiments can be done by combining heating and cooling. Once the required number of frequency shifts is obtained, then, similarly to single mass determination, we again seek positions *h_j_* and the mass ratios *ε*_Σ_ that satisfy Equation (17). However, for multiple attached masses, the most likely attachment positions and, afterwards, the desired attached masses, can be obtained only numerically (see [11] and [46]). The dimensionless sensitivity of NiTi nanocantilever-based mass sensors is given by [11]:

(18) $$S=\frac{\gamma^{2}-\gamma_{\mathrm{add}}^{2}}{\varepsilon }.$$

Interestingly, Equation (18) indicates that the mass sensitivity increases with an increase of *γ*^2^. Hence, for NiTi nanocantilever-based mass sensors, the higher value of *σ*_m_ does not only notably reduce the uncertainties in frequency measurement caused by the temperature fluctuation, but also enhance the mass sensitivity. For instance, for the above-considered NiTi/Si nanocantilever, the adsorbate mass of *ε* = 10^−3^ attached at *h* = 0.8*L* yields for *σ*_m_ (*σ*_a_) = 300 (325) MPa a sensitivity *S* = 7.3 (7.5), whereas, for *σ*_m_ (*σ*_a_) = 500 (525) MPa, the sensitivity is 8.6 (8.8), respectively.

For the second scenario, a uniform material layer formed by the adsorbate molecule on top of the nanocantilever surface causes a variation in both cantilever mass and stiffness. The adsorbate mass shifts the resonant frequencies to lower values, i.e., Δ*f*_add_/*f* = −*m*/(2*M*), where the “−” sign denotes a decrease of the resonant frequency, while its “effective spring (or force) constants” result in an increase of the resonant frequencies; Δ*f*_add_/*f* = *k*/(2*K*), where *k* and *K* are the “spring” constants of the adsorbate and nanocantilever [52,53]. The frequency shift due to adsorbate mass is then a combination of both effects and, in some cases, the adsorbate can cause either an increase of the resonant frequencies or no any detectible frequency shift [54]. For the conventional nanocantilever, its *M* and *K* are given, thus, it is does not, in principle, allow the determination of the adsorbate mass from the measured flexural resonant frequency shifts, i.e., the effect of added mass and stiffness cannot be decoupled. For NiTi nanocantilevers only its mass is given, while its *K* can be intentionally changed by slowly varying the temperature, enabling to decouple the effect of adsorbate mass and stiffness on the cantilever resonant frequencies. For illustration, we suppose the following simple example: two different resonant frequency shifts Δ*f*_add_1_ and Δ*f*_add_2_ caused by adsorbate mass are measured by NiTi nanocantilever of two known different values *K*_1_ and *K*_2_. For a uniform material layer formed on top of the cantilever, the frequency shift due to added *m* and *k* is Δ*f*_add_/*f* = *k*/(2*K*) − *m*/(2*M*). It immediately implies that for *K*_1_ and *K*_2_ the *k* due to adsorbate yields $k=\frac{\left({\Delta f_{\mathrm{add}\text{\_}1}/f}_{1}\right)-\left({\Delta f_{\mathrm{add}\text{\_}2}/f}_{2}\right)}{K_{2}-K_{1}}K_{1}K_{2}$ and, consequently, the desired adsorbate mass can be obtained as $m=2M\frac{\left({\Delta f_{\mathrm{add}\text{\_}1}/f}_{1}\right)K_{1}-\left({\Delta f_{\mathrm{add}\text{\_}2}/f}_{2}\right)K_{2}}{K_{2}-K_{1}}$. Importantly, the theoretical model that explains the frequency response caused by the adsorbate molecule mass and its stiffness bound on an arbitrary location on the cantilever surface is given in [55]. Briefly, the effect of stiffness and mass can be decoupled by positioning the targeted molecule to places where each effect dominates, i.e., mass near the free end and stiffness near the cantilever clamped end. For molecules bound at arbitrary locations on the cantilever, the frequency shift can be calculated by the numerical method, e.g., Raleigh-Ritz [54,55], or analytically by following the approach given in [56] for natural frequencies of a string with an arbitrary number of piece-wise constant mechanical properties.

## 5. Conclusions

Nanocantilevers with significantly changeable Young’s modulus, variable static deflection, and tunable resonant frequencies utilizing a phase transformation of NiTi thin films sputtered on an elastic substrate, such as Si_3_N_4_, Si, or SiO_2_, were explored. The elastic properties of the sputtered NiTi film and the interlayer stress between the film and the substrate varying with temperature were linked to the martensitic phase transformation in the NiTi film. It was shown that the variation of temperature strongly affects the vibrational response of these nanocantilevers. Changes in cantilever static deflection are dominated by variations in the interlayer stress, while the variable elasticity of the NiTi film enables high-frequency tunability of even the cantilever at higher vibrational modes. In contrast to piezoelectric or electrostatic tuning mechanisms, the phase transformation of the NiTi film enables an extraordinarily high-frequency tuning of several tens of percent. It has been shown that errors in the resonant frequency determination caused by uncertainties in temperature can be significantly suppressed, for instance, by using the NiTi nanocantilevers of higher interlayer stress values. It was found that, for common temperature uncertainties, the error in the nanocantilever frequency setup is of the same magnitude (Δ*f* ~ 100 Hz) as is known for the conventional nanocantilevers that utilize the piezoelectric and/or electrostatic effects. Additionally, an application potential for NiTi nanocantilevers in real-time mass spectrometry was demonstrated on two different examples. The results are expected to be of particular value in studies of biomolecule adsorption on nanomechanical sensors [7] including changes in surface elasticity and surface stress, or in future developments of high-frequency operating sensors and actuators with intentionally-changeable properties utilizing smart memory alloys.
